# Processing of Enriched Pear Slices with Blueberry Juice: Phenolics, Antioxidant, and Color Characteristics

**DOI:** 10.3390/antiox12071408

**Published:** 2023-07-11

**Authors:** Siluana Katia Tischer Seraglio, Belkis Sarahí Hernández-Velásquez, Moira Elizabeth Osses-Millar, Bárbara Yolanda Malverde-Muñoz, María Estuardo Guerra-Valle, Constanza Pavez-Guajardo, Jorge Moreno

**Affiliations:** 1 Departamento de Ingeniería en Alimentos, Facultad de Ciencias para el Cuidado de la Salud, Campus Fernando May, Universidad del Bio-Bio, Box 447, Chillán 4081112, Chile; siluanaseraglio@hotmail.com (S.K.T.S.); belkis.hernandez1901@alumnos.ubiobio.cl (B.S.H.-V.); moira.eli31@gmail.com (M.E.O.-M.); barbara.malverde1601@alumnos.ubiobio.cl (B.Y.M.-M.); constanza.pavez.g@gmail.com (C.P.-G.); 2 Departamento de Nutrición y Dietética, Facultad de Ciencias para el Cuidado de la Salud, Campus Concepción, Universidad San Sebastián, Lientur 1457, Concepción 4080871, Chile; maria.guerra@uss.cl

**Keywords:** anthocyanins, ohmic extraction, pulsed-vacuum, *Pyrus communis*, *Vaccinium corymbosum*

## Abstract

This study evaluated the effectiveness of phenolic compound incorporation from blueberry juice into pear slices (PS) using a combination of ohmic heating (OH) and vacuum impregnation (VI), followed by air-drying (AD) or freeze-drying (FD). Our results showed that OH increased the content of bioactive compounds and antioxidant capacity of blueberry juice, with the optimal OH condition set at 50 °C for 20 min under an electric field of 13 V·cm^−1^. Furthermore, the combination of VI and OH was efficient in enriching PS with bioactive compounds from blueberry juice (such as cyanidin and epigallocatechin), with the optimal VI/OH condition set at 50 °C for 90 min under an electric field of 7.8 V·cm^−1^. Moreover, anthocyanin pigments from blueberry juice affected the color parameters of PS by increasing the a* parameter and decreasing the b* and L* parameters. However, both FD and AD (at 40, 50, and 60 °C) negatively affected (*p* ≤ 0.05) the phenolic content and antioxidant capacity. Notably, AD at 60 °C showed the highest levels of phenolic compounds and antioxidant potential for both impregnated and non-impregnated PS.

## 1. Introduction

A diet rich in fruits and vegetables is associated with the minimization of numerous chronic and degenerative diseases, contributing to well-being. Blueberry (*Vaccinium* spp.) and pear (*Pyrus communis* L.) fruits are highly accepted by consumers for their pleasant sensorial characteristics, being consumed fresh and processed as frozen pulps, jams, and jellies. Moreover, they are consumed as sources of phytochemicals with potential beneficial effects on health [1,2,3,4]. Different compounds with nutritional and bioactive potential are present in blueberries and pears, where the phenolic compounds can be highlighted. These compounds are associated with numerous beneficial health properties, including antioxidant, antimicrobial, anti-inflammatory, anti-neurodegenerative, and anticarcinogenic effects, among others [1,3,4,5]. In blueberries, anthocyanins gain special prominence, being present especially in the fruit peel but also in its pulp. These compounds are responsible for the purple color of these fruits and are present in high concentrations in them. Other phenolic compounds in blueberry fruits include phenolic acids, stilbenes, hydrolysable and condensed tannins, and non-anthocyanin flavonoids [3,4,6]. Phenolic compounds are also found in pears, including flavan-3-ols, phenolic acids, flavonols, simple phenolics (hydroquinones), and anthocyanins for colored cultivars [1,2,5].

High perishability of fresh fruits, seasonality, and costs, among other factors, make it challenging to offer fruits in their fresh form constantly. Thus, processing is crucial to providing practical and healthy fruit-based products for immediate and constant consumption. However, changes in physicochemical, sensorial, nutritional, and functional properties can occur in processed products, especially in compounds more susceptible to heat, light, and pH changes. This is the case with phenolic compounds, especially anthocyanins, which can undergo degradation during technological processing [3,7,8,9].

Emerging technological processes such as ohmic heating (OH) and vacuum impregnation (VI) can help obtain fruit-based products enriched with bioactive compounds [8,9,10]. In OH or electroconductive heating, electrodes are in contact with conductive foods that are internally heated by passing an alternating electrical current through them. The presence of ionic constituents such as salts and acids makes most foods conductors of electric current. This technology transforms electrical energy into thermal energy, promoting uniform and rapid food heating. Moreover, the electroporation effect is caused in food by the electric field applied during OH. This effect can break the cell wall and change the food matrix’s structure, facilitating the entry of compounds into cells without necessarily causing physical disruption of the tissue [11,12,13]. OH is especially interesting when associated with VI. In VI, the matrix food is immersed into an impregnation solution (hypertonic, hypotonic, or isotonic), and the vacuum is applied, being released to atmospheric pressure after a time. The hydrodynamic mechanism of pressure changes promotes an air-liquid flow within the food matrix’s pores, in which the impregnation solution will occupy the spaces [8,9,12]. Therefore, when both technological processes are used together (VI/OH), a better mass transfer occurs that accelerates the incorporation of sugars, minerals, vitamins, probiotics, and phenolic compounds, among others, from the impregnation solution into the cellular spaces of the food matrix [8,9,14,15].

Additionally, subsequent application of conventional and emerging technologies such as convection air-drying (AD) and freeze-drying (FD) can contribute to increasing the shelf life of these products by reducing water content and making it possible to expand the range of products offered to the consumer [3,7]. FD is an advanced drying method for obtaining highly sensorial and nutritional products. Due to low temperatures and low pressure to remove water by sublimation, this technique allows the preservation of physical structure and many compounds with low heat stability. However, its operating cost is high and it is rarely used commercially [3,16]. On the other hand, AD is widely applied in the food industry due to its cost-effectiveness. In this process, air passes continuously through food at a given temperature, humidity, and flow rate. However, air-dried foods’ sensorial, nutritional, and bioactive characteristics are more severely affected [3,7,16]. 

Therefore, offering practical and healthy fruit-based products is a constant challenge in the food industry. In this sense, blueberry presents the potential for exploration as a source of bioactive compounds since it is a fruit naturally rich in phenolic compounds, especially anthocyanins, has high acceptability by consumers, has a good yield of juice/pulp, and is suitable for the application of treatments such as OH. On the other hand, pear is an excellent fruit for exploitation as an impregnation matrix, presenting structural characteristics (pores) that favor processes of compound impregnation in addition to being a fruit with soft and pleasant sensorial characteristics, but with a more restricted profile of phenolic compounds. Thus, enriching pear slices (PS) with bioactive compounds from blueberry juice is an attractive alternative to offering new healthy fruit-based products. This study evaluated the kinetics of incorporation and stability of phenolic compounds from blueberry juice treated by OH into PS using VI/OH followed by drying processes (AD and FD). Total phenolic, flavonoid, and anthocyanin contents, antioxidant capacity, individual phenolic compounds, and color parameters were evaluated during this study.

## 2. Materials and Methods

### 2.1. Reagents

Sodium carbonate, Folin–Ciocalteu reagent, potassium chloride, sodium acetate trihydrate, aluminum chloride hexahydrate, sodium hydroxide, sodium nitrite, iron(III) chloride hexahydrate, 2,4,6-tris(2-pyridyl)-s-triazine, Trolox, hydrochloric acid, 2,2-diphenyl-1-picrylhydrazyl (DPPH), ethanol, and HPLC-grade solvents (water, acetonitrile, formic acid, and methanol) were purchased from Merck (Darmstadt, Germany). Phenolic compounds (pelargonin chloride, epigallocatechin, cyanidin chloride, malvidin chloride, petunidin 3-*O*-β-glucosidase chloride, catechin, cyanidin 3-*O*-glucosidase chloride, gallic acid, myricetin, caffeic acid, epigallocatechin gallate, quercetin, *p*-coumaric acid, and epicatechin) were acquired from Sigma-Aldrich (St. Louis, MO, USA). Distilled water was used in the analyses, except for individual phenolic compound determination.

### 2.2. Samples

Blueberries (*V. corymbosum*) and pears (*P. communis* L. cv. Packham’s Triumph) were bought in the Ñuble region, Chile, and cooled at −80 °C and 4 °C, respectively, until their use. Before use, the pears were peeled, cut into 3 × 4 × 0.7 cm slices, and submerged in 2% citric acid and 1% ascorbic acid to prevent oxidation [9]. 

### 2.3. Blueberry Treatment by OH

Whole and thawed blueberries were added to the water in the proportion of 1:1.2 (*w/w*, fruit: water), and an electric field strength of 13 V·cm^−1^ at 30, 40, and 50 °C for 5, 10, 15, and 20 min was applied. The electric field strength was calculated as the voltage/electrode distance ratio. OH equipment consisted of two concentric stainless-steel cylinders (3.7 and 19 cm in diameter) with a plastic bottom connected to a power source by two electrodes. As the applied electric field strength was constant throughout the experiment, the three temperatures evaluated were achieved using a thermoregulated bath and orbital shaker (Barnstead/Lab-line MaxQ 2000, Champaign, IL, USA). After OH treatment, blueberries were pressed and filtered to obtain the juice. Blueberry juice was kept at 4 °C in the dark in the absence of oxygen for posterior use. The best OH treatment was selected using the total bioactive compounds and antioxidant capacity data. 

### 2.4. VI/OH of PS with Blueberry Juice

VI/OH treatment was applied with minor modifications, according to Pavez-Guajardo et al. [8]. VI treatment was carried out at 50 mbar for 5 min in PS added to impregnation solution in the proportion of 1:3 (*w/w*, pear: solution). Blueberry juice obtained by the selected OH treatment was used as the impregnation solution. In the sequence, the impregnation process was completed by applying OH with an electrical field strength of 7.8 V·cm^−1^ (alternating current at 60 Hz and 60 V) at 30, 40, and 50 °C for up to 180 min. As the applied electric field strength was constant throughout the experiment, the three temperatures evaluated were achieved using a thermoregulated bath and an orbital shaker. The best VI/OH treatment of PS with blueberry juice was selected according to the total bioactive compounds and antioxidant capacity data.

### 2.5. Drying Processes of PS with Blueberry Juice

**Figure 1:**
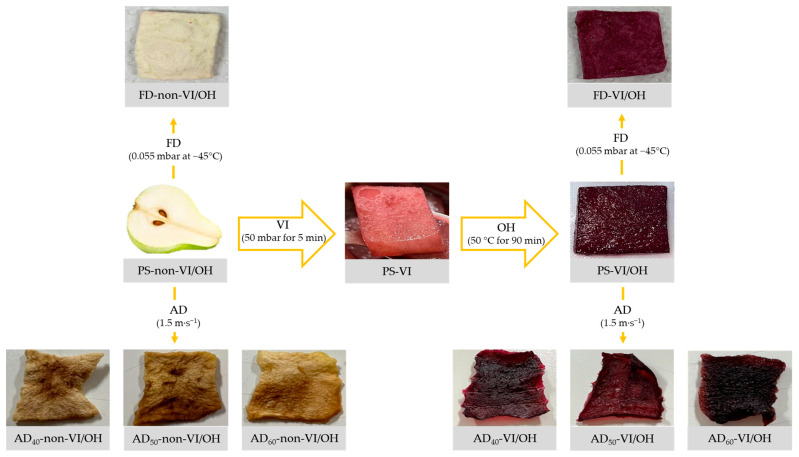
The process applied to obtain the pear slices (PS) impregnated by vacuum impregnation (VI), followed by ohmic heating (OH), and dried and non-dried by freeze-drying (FD) and air-drying (AD): PS treated by VI/OH and non-dried (PS-VI/OH) and dried by freeze-drying (FD-VI/OH) and air-drying at 40, 50, and 60 °C (AD_40_-VI/OH, AD_50_-VI/OH, and AD_60_-VI/OH). Control samples: PS non-treated by VI/OH and non-dried (PS-non-VI/OH) and dried by freeze-drying (FD-non-VI/OH) and air-drying at 40, 50, and 60 °C (AD_40_-non-VI/OH, AD_50_-non-VI/OH, and AD_60_-non-VI/OH).

New PS and blueberry juice samples were obtained for the drying study to prevent the early degradation of bioactive compounds. Drying of impregnated/non-impregnated PS samples was evaluated by AD and FD. AD was carried out in a forced air oven (Memmert, Model HCP-108, Schwabach, Germany) at 40, 50, and 60 °C and with an air velocity of 1.5 m·s^−1^. FD was carried out at −45 °C and 0.055 mbar in a manual freeze dryer (Labconco, Freezone 2.5 manual, Kansas, MO, USA). Drying processes occurred until the samples reached 18% moisture, which was monitored by controlling the weight of the samples [17]. Figure 1 shows the process applied to obtain the PS samples evaluated in this study.

### 2.6. Total Bioactive Compounds and Antioxidant Capacity

Triturated samples (5 g) of impregnated/non-impregnated and dried/non-dried PS samples were extracted with 10 mL of ethanol:water (6:4, *v/v*) for 20 min in a shaker (Gerhardt, model RO-500, Königswinter, Germany) protected from light. The supernatant was collected after centrifugation for 10 min at 10 °C and 4000 rpm. The residue was subjected to the extraction process twice more, and the supernatants were combined. Blueberry juice was analyzed directly. 

Total phenolic content (TPC) was determined using the Folin–Ciocalteu method described by Waterhouse [18], and the results were expressed as mg of gallic acid equivalent (GAE) per 100 mL or 100 g of dry matter (DM). Total flavonoid content (TFC) was determined using the colorimetric method described by Dewanto et al. [19], and the results were expressed as mg of catechin equivalent (CE) per 100 mL or 100 g of DM. Total monomeric anthocyanin content (TMA) was determined using the differential pH method described by Lee et al. [20], with minor modifications, and the results were expressed as µg cyanidin 3-glucoside equivalent (C3G) per 100 mL or 100 g of DM. Detailed information is presented in Table S1.

DPPH free radical scavenging assay was performed according to the method reported by Thaipong et al. [21], with minor modifications, and the results were expressed as µmol Trolox equivalents (TE) per 100 mL or 100 g of DM. Ferric reducing antioxidant power (FRAP) assay was performed according to Benzie and Strain [22], with minor modifications [21], and the results were expressed as µmol TE per 100 mL or 100 g of DM. Detailed information is presented in Table S1.

### 2.7. Color Parameters

The color was measured in a spectrocolorimeter (Konica Minolta Optics Inc., CM-5, Tokyo, Japan) adjusted to operate with a D65 illuminant, an observer angle of 10°, and in reflectance mode. CIE L* (darkness–lightness), a* (green–red axis), and b* (blue–yellow axis) coordinates were measured against a white background.

### 2.8. Phenolic Compounds by High-Performance Liquid Chromatography (HPLC)

Three solvent systems (water, ethanol:water (6:4, *v/v*), and methanol:water (8:2, *v/v*)) were evaluated for the extraction of phenolic compounds from dried and impregnated/non-impregnated PS samples, and the methanol system obtained the best signal/noise ratio. Briefly, triturated samples were extracted with methanol:water (8:2, *v/v*) in the proportion of 1:2, respectively, for 1 h in an ultrasonic bath (Elma, E30H Elmasonic, Singen, Germany) at 10 °C and protected from light. After centrifugation for 20 min at 10 °C and 7000 rpm, the supernatant was collected and filtered using 45 µm PTFE syringe tip filters (Merck Millipore, Dublin, Ireland) before injection on the HPLC system. For the non-dried and impregnated/non-impregnated PS samples, triturated samples were centrifugated for 20 min at 10 °C and 7000 rpm, and the supernatant was filtered before injection. Blueberry juice was analyzed after filtration.

Fourteen phenolic compounds were determined according to the method described by Ruiz et al. [23], with minor modifications, using a series 200 HPLC system from Perkin Elmer (Waltham, MA, USA) coupled to a diode array detector (more information in Table S1). Results were expressed as mg per 100 mL or 100 g of DM.

### 2.9. Statistical Analysis 

Data were presented as the mean ± standard deviation of three independent replicates. Statistical differences between mean values were verified by the analysis of variance (ANOVA) followed by the least significant difference (LSD) test using the statistical program Statgraphics Centurion XVI Software (version 16, Statgraphics Technologies Inc., The Plains, VA, USA). All statistical tests were performed at a 5% level (*p* ≤ 0.05).

## 3. Results and Discussion

### 3.1. Blueberry Treatment by OH

Blueberry fruits contain several bioactive compounds, among which anthocyanins can be highlighted. These compounds are responsible for their purple color and may represent more than 50% of the total content of phenolic compounds in these fruits. However, other phenolic groups are also present in blueberries, such as phenolic acids and non-anthocyanin flavonoids [4,6]. An efficient transfer of phenolic compounds and other bioactive compounds to products and raw materials obtained from blueberries, such as blueberry juice, is essential to enriching these products with compounds with potential health effects. OH can assist in this regard, as it promotes the electroporation effect, stimulating the break of the cell wall and facilitating subsequent extraction of compounds of interest without causing physical disruption of the food matrix [13,24,25].

**Figure 2:**
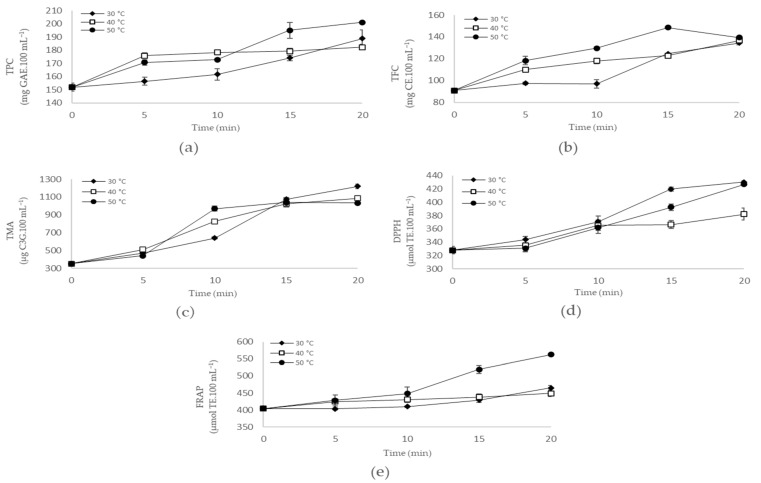
Effect of ohmic heating on blueberry juice throughout 20 min at 30, 40, and 50 °C: (**a**) total phenolic content (TPC); (**b**) total flavonoid content (TFC); (**c**) total monomeric anthocyanin content (TMA); (**d**) DPPH (2,2-diphenyl-1-picrylhydrazyl) free radical scavenging assay; and (**e**) ferric reducing antioxidant power (FRAP). GAE—gallic acid equivalent. CE—catechin equivalent. C3G—cyanidin 3-glucoside equivalent. TE—Trolox equivalent. DM—dry matter. Results are presented as mean ± standard deviation.

In this study, whole blueberry fruits were submitted to OH, aiming especially for the disruption of cell walls from peel fruit to facilitate the extraction of anthocyanins since these compounds are present in high amounts in this fruit part [4,26]. As shown in Figure 2 (and Table S2), the values of TPC, TFC, and TMA of blueberry juice were affected by the different OH treatments. In general, the increase in the time and temperature of OH promoted an increase in the values of TPC, TFC, and TMA of blueberry juice, indicating better efficiency in the rupture of the cell wall and, consequently, in the accessibility of bioactive compounds to be extracted from the fruit. The highest values for TPC (201.09 mg GAE.100 mL^−1^) and TFC (148.57 mg CE.100 mL^−1^) were observed in OH treatment at 50 °C for 20 and 15 min, respectively. The highest value for TMA was found in OH treatment at 30 °C for 20 min (1219.04 µg C3G.100 mL^−1^), although high values for TMA were also observed at 40 and 50 °C for 15 and 20 min. These findings indicate that OH contributes to the extraction of phenolic compounds from blueberry fruit but may negatively affect anthocyanin content. Despite being potent antioxidant compounds, anthocyanins have low stability, influenced by many factors such as moisture, light, pH, temperature, oxygen, sugar content, metals, and enzymes [27,28]. Therefore, our data suggest that longer times and temperatures of OH are unlikely to promote intense improvements in the extraction of anthocyanins, which may favor the degradation of this critical group of phenolic compounds in blueberries.

Phenolic compounds are well known due to their beneficial health properties, among which antioxidant capacity can be highlighted. Compounds with potential antioxidant action against the oxidation process can act through different mechanisms, which include scavenging free radicals, chelating metal ions, quenching singlet oxygen and secondary oxidation products, and inactivating reactive oxygen and nitrogen species [29,30]. Through antioxidant action, these compounds attenuate the effects of oxidative stress and minimize other harmful health effects. They also have neuroprotective, anti-inflammatory, anticancer, and cardioprotective effects, among others [26,29]. As observed for TPC, TFC, and TMA, the different OH treatments also affected the antioxidant capacity of blueberry juice (Figure 2 and Table S2). Especially for the FRAP assay, increasing the time and temperature of OH increased the antioxidant capacity of the OH-treated blueberry juices. The highest values for the DPPH assay were found in OH at 30 °C for 15 and 20 min and at 50 °C for 20 min (419.88 to 430.16 µmol TE.100 mL^−1^), while for the FRAP assay, the highest value was verified at 50 °C for 20 min (562.24 µmol TE.100 mL^−1^). These findings seem related to the content of phenolic compounds extracted from blueberry fruit in each OH condition studied. For example, the high value for DPPH at 30 °C for 15/20 min can be linked to the TMA of blueberry juice, as can the high values for DPPH and FRAP at 50 °C for 15/20 min, which are possibly associated with the values of TPC and TFC, besides TMA. The increase in the values of TPC and antioxidant capacity by the FRAP assay was also reported for carrot and mulberry juices treated by OH and associated with the electroporation effect that facilitates the release of phenolic compounds with antioxidant potential [31,32,33].

Therefore, OH proves to be an essential technological tool to improve the bioactive compound content of fruit juices. Considering the data obtained, the OH treatment selected to obtain the blueberry juice was set at 50 °C for 20 min, during which high values of TPC, TFC, TMA, and antioxidant capacity were obtained.

### 3.2. VI/OH of PS with Blueberry Juice

The search for functional foods that contain constituents with potential beneficial health effects is constant. Thus, the enrichment of foods with bioactive compounds was promising and applied in this study. Pears are fruits with high consumer acceptance and contain sugars, fibers, organic acids, minerals, amino acids, fatty acids, and phenolic compounds such as phenolic acids and flavonoids [2,34]. However, the presence of anthocyanins is limited to cultivars with purple skin, such as the anthocyanins petunidin (cv. Winter Nelis) and cyanidin (cv. Beurre Bosc) [35]. In this sense, enriching PS with blueberry juice can improve the content of phenolic compounds, especially the anthocyanins, and provide food with increased functional potential. The VI technique is an excellent tool for incorporating constituents such as phenolic compounds into porous foods, such as pears. Due to the pressure changes that occur during VI, the pores in the food matrix are filled with the impregnation solution, thus ensuring the incorporation of constituents into this matrix [9,15,36]. The assistance of VI with emerging technologies such as OH, ultrasound, and pulsed electric field [37,38,39] can improve the enrichment of the food matrix and mass transfer. In the case of associating VI with OH, the incorporation of compounds can be accelerated due to the electroporation effect caused by OH, which can facilitate the entry process of impregnation solution into the pores of the food matrix [8,15].

**Figure 3:**
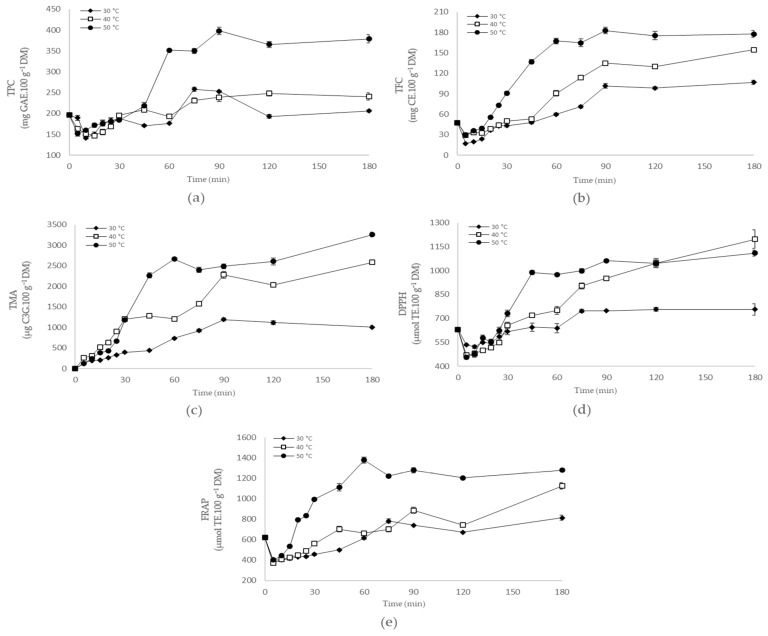
Effect of vacuum impregnation assisted by ohmic heating on pear slices enriched with blueberry juice throughout 180 min at 30, 40, and 50 °C: (**a**) total phenolic content (TPC); (**b**) total flavonoid content (TFC); (**c**) total monomeric anthocyanin content (TMA); (**d**) DPPH (2,2-diphenyl-1-picrylhydrazyl) free radical scavenging assay; and (**e**) ferric reducing antioxidant power (FRAP). GAE—gallic acid equivalent. CE—catechin equivalent. C3G—cyanidin 3-glucoside equivalent. TE—Trolox equivalent. DM—dry matter. Results are presented as mean ± standard deviation.

In Figure 3 (and Table S3), it is possible to verify that VI/OH treatment indeed increased the phenolic compounds’ content on PS-VI/OH samples (PS treated by VI/OH and non-dried). For all temperatures studied, the values of TPC and TFC decreased in the first minutes of VI/OH treatment compared to the PS-non-VI/OH sample (PS non-treated by VI/OH and non-dried). It was associated with the effects of VI treatment on the pear matrix, which may have caused, for example, the initial departure of constituents from the matrix into the impregnation solution to establish a balance in the medium. However, with increasing OH, the values of TPC and TFC increased up to around 90 min, during which they maintained some degree of stability up to 180 min for all temperatures studied. Furthermore, impregnation was more efficient as the temperatures of OH increased, and the highest values of TPC and TFC were obtained at 50 °C per 90 min (398.03 mg GAE.100 g^−1^ DM and 182.62 mg CE.100 g^−1^ DM, respectively).

As the PS-non-VI/OH sample did not contain anthocyanins, VI treatment itself promoted the impregnation of color compounds into PS, which was intensified with the subsequent application of OH (Figure 1 and Figure 3, and Table S3). As shown in Figure 3 (and Table S3), anthocyanin impregnation was higher as the temperature of OH increased, as well as with the increase in the time of OH up to around 90 min, which showed relative stability up to 180 min. However, the highest TMA value was observed at 50 °C (3258.69 µg C3G.100 g^−1^ DM). Despite this, it is essential to highlight that, based on our data, more extended times of OH would not promote intense increases in TMA values once the plateau was reached. At the same time, higher temperatures of OH would possibly favor the degradation of anthocyanins and not promote an intense increase in the impregnation process, as discussed in the OH treatment of blueberry juice. Therefore, the association of OH with VI proved essential to ensure an adequate enrichment of PS with bioactive compounds, especially anthocyanins.

Enrichment of PS with bioactive compounds promoted by the VI/OH treatments consequently affected their antioxidant capacity, as shown in Figure 3 (and Table S4). As verified for TPC and TFC, a decrease in the values for DPPH and FRAP was observed in the first minutes of VI/OH treatment compared to the PS-non-VI/OH sample and linked to the effects of VI treatment on the pear matrix. However, for all temperatures studied, increasing the time of OH promoted an increase in the values for DPPH and FRAP, especially up to around 90 min, although the highest values were observed at 180 min in many cases. Furthermore, the impregnation of antioxidant compounds showed to be more efficient as the temperatures of OH increased. In a previous study, VI/OH intensified the mass transfer in osmodehydrated blueberries (65%, *w/w*, sucrose solution) treated at 30, 40, or 50 °C for 300 min using an electric field of 13 V·cm^−1^. The intermediate temperature (40 °C) was selected for VI/OH treatment since it provided the best balance between mass transfer (greater at higher temperatures) and retention of phenolic compounds (greater at lower temperatures) [10]. In our study, the best retention rates of phenolic compounds and antioxidant capacity were generally observed at the highest temperature evaluated (50 °C). This behavior seems to suggest that the retention of bioactive compounds depends on the compositional characteristics of each food system (e.g., high sugar or low sugar content), which are affected by factors such as temperature. Therefore, these findings demonstrate the need to study the most suitable conditions for each technological process applied both for blueberry juice extraction (by OH) and for the enrichment of PS with blueberry juice (by VI/OH) since the food matrices differ. Thus, the results obtained from technological processes can also be different. 

The antioxidant capacity of PS-VI/OH samples seems to be strongly associated with the content of bioactive compounds impregnated in the different conditions studied. Our findings suggest that non-anthocyanidin antioxidant compounds, such as phenolic acids and non-anthocyanin flavonoids, are the compounds primarily responsible for the antioxidant capacity of PS-VI/OH samples, with an increasing contribution of anthocyanins to this activity as temperature and time of OH increase. This fact leads, in general, to higher values of antioxidant capacity being observed after 90 min of VI/OH treatment, especially at 180 min, where the highest TMA values were observed for the temperature conditions of 40 and 50 °C.

Therefore, the association of OH with VI improved the bioactive compound content of PS. Considering the data obtained, the VI/OH treatment selected to obtain PS enriched with blueberry juice was set at 50 °C for 90 min, during which high values of TPC, TFC, TMA, and antioxidant capacity were obtained.

### 3.3. Drying Processes of PS with Blueberry Juice

The seasonality of production, short shelf life, accessibility, and cost of the product in the market commonly limit the availability of fresh fruits. Thus, processed products present great convenience to the consumer because the product will constantly be available and is generally suitable for immediate consumption [3,7,8,9]. Technological processes such as AD, FD, and osmotic dehydration can be applied to products from fruits to improve their conservation, as they promote the reduction in water content and concentration of solutes, mainly when heated, and encourage microbial decline [3,7,8,9,10]. This set of factors allows an extension of the shelf life of processed products compared to fresh ones, in addition to other advantages such as storage at room temperature and reduction of transport and storage costs, among others. On the other hand, physicochemical, sensorial, nutritional, and functional changes can occur in processed products, especially considering compounds are more susceptible to heat, light, and pH changes, among other altered factors in processed products. One example is the phenolic compounds, especially anthocyanins, which can undergo degradation when applying technological processes [3,7,8,9].

#### 3.3.1. Enriched PS with Blueberry Juice before Drying Processes

Considering the non-dried PS, it was observed that, as expected, the process of impregnating PS with blueberry juice by VI/OH promoted an increase in the values of TPC, TFC, TMA, and antioxidant capacity of the PS-VI/OH sample compared to the PS-non-VI/OH sample (Table 1), indicating the enrichment of this product with bioactive compounds. As shown in Table 2, blueberry juice has several phenolic compounds in its composition, therefore being an impregnation solution with a high potential for enrichment of other food matrices. Anthocyanins pelargonin, cyanidin, petunidin 3-glucosidase, and cyanidin 3-glucosidase were quantified in blueberry juice, ranging from 62.22 to 3.24 mg.100 mL^−1^. In addition to anthocyanins, five non-anthocyanin flavonoids (epigallocatechin, catechin, epicatechin, myricetin, and quercetin) and three phenolic acids (gallic, caffeic, and *p*-coumaric acids) were also quantified in blueberry juice, in which epigallocatechin and epicatechin stood out with the highest concentrations (125.47 and 30.81 mg.100 mL^−1^, respectively).

In general, it is possible to observe an increase in the concentration of phenolic compounds in the PS-VI/OH sample (Table 2) compared to the PS-non-VI/OH sample, indicating that this food was indeed enriched with bioactive compounds and in agreement with the findings found in Table 1. Incorporating anthocyanins into PS was visibly verified (Figure 1), promoting changes in the color parameters (Table 1). The presence of anthocyanin pigments from blueberry juice in PS promoted an increase in the value of a* (trend towards red coloration) and a reduction in the values of b* (trend towards blue color) and L* (tendency towards darkness) of the PS-VI/OH sample compared to the PS-non-VI/OH sample. Color changes are due to the retention of these pigments from blueberry juice in the pores of the pear matrix [8,15]. However, as shown in Table 2, only cyanidin was quantified in the PS-VI/OH sample. This may be due to the lower stability of other anthocyanins quantified after the VI/OH process since anthocyanins are more susceptible to degradation [27,28], associated with lower susceptibility to impregnation in the studied matrix. In addition, color compounds from the degradation of anthocyanins and other anthocyanins not quantified in this study may also have contributed to the color and bioactive potential of the PS-VI/OH sample. In agreement with our findings, in the study conducted by Guerra-Valle et al. [9] with apple slices osmodehydrated with 47 °Brix pomegranate cryoconcentrated juice and treated by VI/OH at 30, 40, or 50 °C for 180 min with an electric field of 6.66 V·cm^−1^, it was observed that VI/OH was able to increase the concentration of bioactive compounds in relation to the fresh sample, especially increasing the values of TMA at 50 °C. These data reinforce the potential of VI/OH as a combined technique for the enrichment of plant matrices, including by using solutions naturally rich in bioactive compounds, such as the blueberry juice treated by OH used in this study.

The impregnation process also contributed to the increase in the concentration of epigallocatechin, catechin, and myricetin in the PS-VI/OH sample compared to the PS-non-VI/OH sample. On the other hand, a decrease in the concentration of gallic acid, caffeic acid, and epicatechin was observed in the PS-VI/OH sample compared to the PS-non-VI/OH sample (Table 2). These findings indicate the migration of bioactive compounds between blueberry juice and pear in equilibrium processes, contributing to the increase of some compounds and the decrease of others. However, in general, it is possible to consider that the impregnation process promoted an increase in the concentration of bioactive compounds in PS (sum of quantified phenolic compounds of 243.68 mg.100 g^−1^ DM for PS-non-VI/OH sample and 462.49 mg.100 g^−1^ DM for PS-VI/OH sample), which consequently impacted its bioactive potential.

Therefore, the enrichment of PS with bioactive compounds from blueberry juice by VI/OH makes it possible to offer a fruit-based product with improved bioactive potential compared to the non-impregnated product. However, the water content of the PS-VI/OH sample and the PS-non-VI/OH sample (81.0 and 82.0%, respectively) is an important limiting factor for their shelf life. Thus, applying the technological processes of FD and AD can promote a reduction in water content and consequently increase the product’s shelf life [3,7]. 

#### 3.3.2. Enriched PS with Blueberry Juice after Drying Processes

In this study, samples were dried until reaching 18% moisture. Although the extension of their shelf life is assured in this condition, the applied drying processes promoted intense alteration of bioactive and color characteristics for both non-impregnated and impregnated PS samples, as shown in Table 1 and Table 2.

Considering the dried PS, it is possible to observe that all dying conditions promoted an intense decrease in the values for TPC, TFC, TMA, and antioxidant capacity compared to non-dried PS (Table 1). This same behavior was observed for phenolic compounds, except for quercetin, for which significant decreases were observed for dried PS compared to non-dried PS, reaching reductions of up to 4.8 and 7.1-fold in the sum of quantified phenolic compounds for non-impregnated and impregnated PS samples, respectively (Table 2). Phenolic compounds comprise a diverse range of compounds with different chemical structures. They can be degraded to varying levels by factors such as light, oxygen, oxidizing compounds, metals, temperature, pH, and enzymes, to mention a few [40,41,42,43]. Therefore, drying processes involve a set of factors with a high potential for phenolic compound degradation, such as light, heat, oxygen, and exposure time, which act together with characteristics of the product, such as pH, enzymes, metals, oxidizing compounds, and water, among others, and can enhance the degree of degradation of phenolic compounds [40,44]. For example, it is known that factors such as pH, moisture, oxygen, heat, and biotransformation (microbial metabolism), among others, can act together or separately on non-gallate and gallate-type catechins (such as catechin, epicatechin, epigallocatechin, and their gallate esters), promoting their degradation or polymerization with the formation of new compounds [45,46,47]. Many studies have been conducted with epigallocatechin gallate, suggesting that its degradation, especially via oxidative reactions, is stimulated in aqueous solutions at low temperatures (40–50 °C). In contrast, at higher temperatures, the epimerization reactions are facilitated by forming epigallocatechin gallate from gallocatechin gallate and gallocatechin gallate from epigallocatechin gallate [45,47]. In the study carried out by Zeng et al. [47], the authors observed that although sunlight and especially oxygen stimulated the degradation of epigallocatechin gallate, its degradation also occurred in systems free from these factors, indicating that the degradation of epigallocatechin gallate occurs independently of auto-oxidation and is possibly associated with epimerization processes. Sang et al. [48] observed that gallocatechin gallate was the primary metabolite produced from epigallocatechin gallate at high concentrations and low auto-oxidation conditions. In contrast, the formation of epigallocatechin gallate dimers was favored when low concentrations of epigallocatechin gallate were applied. Therefore, it is possible to suggest that the reduction in the concentration of epigallocatechin, the main phenolic compound quantified in impregnated and non-impregnated PS samples, observed in dried PS samples is possibly associated with a set of reactions, in particular via oxidative and epimerization, stimulated by factors such as heat, oxygen, light, and concentration, with epigallocatechin dimers, gallocatechin, as well as oxidative products as possible secondary metabolites [47,48,49].

The increase in quercetin concentration can also be considered a result of the degradation/hydrolysis processes of phenolic compounds present in impregnated and non-impregnated PS samples caused by drying. The presence of glycosides of quercetin such as rutin (quercetin 3-*O*-rutinoside), isoquercitrin (quercetin 3-β-d-glucoside), quercitrin (quercetin 3-rhamnoside), hyperoside (quercetin 3-galactoside), and miquelianin (quercetin 3-*O*-glucuronide) has already been reported in blueberry and pear fruits, these compounds being susceptible to hydrolysis with consequent release of aglycone quercetin [4,6,50,51]. In this sense, our data suggest that drying processes promoted the degradation at different levels of individual phenolic compounds present in both impregnated and non-impregnated PS samples, consequently affecting the total content of bioactive compounds and antioxidant potential of dried PS samples compared to non-dried PS samples. Besides that, it is possible to observe different levels of influence for each drying condition on phenolic compounds, antioxidant capacity, and color parameters.

##### AD and FD effects on Enriched PS

As shown in Table 1, among the drying conditions for non-impregnated PS samples, more intense decreases were observed in the values of TPC, TFC, DPPH, and FRAP for AD at 40 and 50 °C (up to 9.6, 46.6, 24.8, and 23.5-fold, respectively, compared to PS-non-VI/OH sample) and FD (26.4, 70.0, 27.3, and 24.9-fold, respectively, opposed to PS-non-VI/OH sample) compared to AD at 60 °C (4.4, 12.6, 6.6, and 6.0-fold, respectively, in contrast to PS-non-VI/OH sample). Considering the drying conditions for impregnated PS samples, the same behavior was observed for TMA, DPPH, and FRAP values, in which more intense decreases were observed for AD at 40 and 50 °C (up to 5.3, 8.4, and 7.7-fold, respectively, compared to the PS-VI/OH sample) and FD (4.2, 7.6, and 7.0-fold, respectively, opposed to the PS-VI/OH sample) compared to AD at 60 °C (3.3, 4.7, and 4.3-fold, respectively, in contrast to the PS-VI/OH sample). For TPC, the highest decrease was verified for FD (6.3-fold compared to the PS-VI/OH sample), while the lowest reduction was obtained for AD at 50 °C (2.5-fold in contrast to the PS-VI/OH sample). On the other hand, FD promoted the lowest TFC value (6.6-fold opposed to the PS-VI/OH sample), while the highest decrease was observed for AD at 40 and 50 °C (up to 9.9-fold in relation to the PS-VI/OH sample). In osmodehydrated blueberries pre-treated by VI/OH (40 °C, 240 min, electric field of 13 V·cm^−1^) and AD at 50, 60, or 70 °C, it was established that drying at 60 °C was the best condition, allowing a good balance between the retention of bioactive compounds and drying time [10]. Therefore, our findings corroborate data from the literature, in which the AD at 60 °C seems adequate for obtaining dry products with good retention of bioactive compounds.

Considering the individual phenolic compounds, it is possible to observe that the drying conditions affected each analyte differently (Table 2). Although there was no significant difference between the drying conditions studied, a trend toward lower degradation of cyanidin after FD and higher degradation after AD at 60 °C was observed. For myricetin and gallic acid, it is also possible to maintain a trend of higher degradation of these phenolic compounds as the temperature of the AD increases. Moreover, the values found for AD conditions were significantly lower than those after FD for myricetin (up to 1.4-fold for impregnated PS samples). At the same time, they were generally statistically equal to those found after FD for gallic acid.

In contrast to these findings, a trend of increasing concentration with increasing AD temperature was observed for epigallocatechin, epicatechin, and caffeic acid. The values found for AD conditions were significantly higher than those seen after FD for epigallocatechin (up to 3.6 and 2.9-fold for non-impregnated and impregnated PS samples, respectively). At the same time, they were considerably lower than those found after FD for epicatechin (up to 1.4 and 1.7-fold for non-impregnated and impregnated PS samples, respectively). For caffeic acid, the values found for AD conditions were statistically equal to those verified after FD. A significantly higher concentration of catechin after AD at 60 °C was also observed compared to the other drying conditions (up to 1.3 and 1.4-fold for non-impregnated and impregnated PS samples, respectively), as well as similar concentrations for the other drying conditions. A significant increase in quercetin concentration was observed with increasing AD temperature. Moreover, the values found for FD were significantly lower than those found for AD conditions at 50 and 60 °C (up to 1.6 and 1.4-fold for non-impregnated and impregnated PS samples, respectively).

Based on these data, it is possible to suggest that changes in the concentration of the investigated phenolic compounds are probably linked to processes of degradation and hydrolysis of phenolic compounds stimulated during drying processes. The extrinsic factors involved in the drying conditions (such as light, oxygen, and heat) and the intrinsic factors associated with the food product (such as enzymes, water, sugars, and pH) can stimulate the degradation of phenolic compounds [42,52], leading to the decrease observed in the concentration of cyanidin, gallic acid, and myricetin. At the same time, the increase in quercetin concentration may be associated with the hydrolysis of quercetin glycosides, as well as the increase in epigallocatechin, catechin, and epicatechin concentrations, which may be related to the hydrolysis of condensed tannins (proanthocyanidins), thus releasing the quercetin, epigallocatechin, catechin, and epicatechin aglycones [53,54]. The release of phenolic compounds with a more straightforward mechanism can be interesting from a biological point of view since gastrointestinal absorption is favored for phenolic compounds with low molecular weight. Therefore, phenolic compounds with a complex structure (such as anthocyanins, non-anthocyanin flavonoids, and tannins) have low bioavailability compared to phenolic compounds with a simple structure (such as phenolic acids and aglycones of non-anthocyanin flavonoids and anthocyanins) [55,56,57]. However, they may also be more susceptible to degradation during product storage and gastrointestinal digestion [42,57]. Therefore, the applied drying processes will provide products with different degrees of phenolic composition and antioxidant potential, and AD at 60 °C seems to give a product with high levels of phenolic compounds and antioxidant potential for both impregnated and non-impregnated PS samples. 

The applied drying processes also promoted changes in the samples’ physical and color characteristics (Table 1 and Figure 1). Dried PS with AD showed shape, volume, and texture changes, as expected when heat is used. Moreover, the influence of AD on the color of samples was observed mainly for non-impregnated PS samples, where a certain degree of browning seems to have occurred, probably due to non-enzymatic browning reactions [3,7,26]. For these samples, a decrease in luminosity (L*) and a tendency towards red and yellow coloration (increase in the values of a* and b*) were verified compared to the PS-non-VI/OH sample. The impregnated and dried PS samples maintained color parameters very close to those observed for the PS-VI/OH sample. The PS samples dried by FD showed a porous texture, and their shape and volume were preserved but with reduced weight, characteristics expected for this technological process [3,14]. For the FD-non-VI/OH (PS non-treated by VI/OH and FD) sample, a decrease in luminosity (L*) and a tendency towards red and yellow coloration (increase in the values of a* and b*) were observed in contrast to the PS-non-VI/OH sample. However, there was an increase in the parameters L*, a*, and b* for the FD-VI/OH (PS treated by VI/OH and FD) sample compared to the values found for the PS-VI/OH sample, which suggests a possible loss of color compounds to some degree.

## 4. Conclusions

In this study, it was possible to confirm that OH contributes to the increase in the content of bioactive compounds and antioxidant potential of blueberry juice, making this product an essential source of bioactive compounds for the enrichment of vegetable matrices, such as pears. OH at 50 °C for 20 min with an electrical field of 13 V·cm^−1^ was chosen as the best treatment for blueberry juice. Furthermore, the VI associated with the OH ensured the enrichment of pear pieces with bioactive compounds from blueberry juice, increasing the concentration of bioactive compounds in the pear, such as cyanidin, epigallocatechin, and catechin, which consequently impacted the pear’s bioactive potential and color parameters. VI/OH at 50 °C for 90 min with an electrical field of 7.8 V·cm^−1^ was selected as the best treatment for impregnating PS with blueberry juice. However, when FD and AD processes were applied, the content of phenolic compounds and the antioxidant capacity decreased for both impregnated and non-impregnated PS samples, in addition to impacting the color parameters. Among the drying processes, the AD at 60 °C seems to provide a product with high levels of phenolic compounds and antioxidant potential.

## Figures and Tables

**Table 1:** Total bioactive compounds, antioxidant capacity, and color parameters of pear slices (PS) impregnated and non-impregnated with blueberry juice before and after drying processes.

Sample	TPC (mg GAE.100 g^−1^ DM)	TFC (mg CE.100 g^−1^ DM)	TMA (µg C3G.100 g^−1^ DM)	Antioxidant Capacity		Color Parameters		
				DPPH (µmol TE.100 g^−1^ DM)	FRAP (µmol TE.100 g^−1^ DM)	L*	a*	b*
PS-non-VI/OH	228.03 ± 5.74 ^g^	84.76 ± 5.31 ^e^	-	838.42 ± 8.79 ^d^	876.34 ± 12.54 ^d^	76.16 ± 2.75 ^e^	−3.36 ± 0.10 ^a^	17.86 ± 0.56 ^d^
AD_40_-non-VI/OH	24.16 ± 1.25 ^abc^	3.42 ± 0.30 ^ab^	-	35.27 ± 1.24 ^a^	38.24 ± 1.12 ^a^	48.23 ± 1.51 ^c^	13.17 ± 0.40 ^e^	32.86 ± 1.43 ^fg^
AD_50_-non-VI/OH	23.82 ± 4.43 ^ab^	1.82 ± 0.08 ^a^	-	33.74 ± 1.52 ^a^	37.25 ± 1.53 ^a^	46.33 ± 1.93 ^c^	14.28 ± 0.65 ^f^	32.58 ± 1.36 ^f^
AD_60_-non-VI/OH	52.17 ± 2.45 ^cd^	6.74 ± 0.34 ^b^	-	127.36 ± 3.35 ^b^	145.25 ± 5.36 ^b^	38.33 ± 1.98 ^b^	13.59 ± 0.78 ^ef^	26.33 ± 1.44 ^e^
FD-non-VI/OH	8.65 ± 0.24 ^a^	1.21 ± 0.04 ^a^	-	30.73 ± 1.03 ^a^	35.23 ± 1.35 ^a^	70.43 ± 2.66 ^d^	7.75 ± 0.44 ^d^	34.40 ± 1.67 ^g^
PS-VI/OH	299.90 ± 7.50 ^h^	120.26 ± 5.43 ^f^	6290.02 ± 100.23 ^d^	1031.85 ± 14.42 ^e^	1097.43 ± 19.42 ^e^	20.57 ± 0.83 ^a^	6.16 ± 0.35 ^c^	1.43 ± 0.09 ^ab^
AD_40_-VI/OH	67.33 ± 3.83 ^de^	12.73 ± 0.51 ^c^	1690.27 ± 50.24 ^bc^	136.73 ± 2.35 ^b^	153.45 ± 5.24 ^b^	18.86 ± 0.94 ^a^	2.08 ± 0.10 ^b^	1.32 ± 0.09 ^a^
AD_50_-VI/OH	118.46 ± 6.83 ^f^	12.14 ± 0.45 ^c^	1180.53 ± 30.74 ^a^	123.27 ± 3.69 ^b^	142.53 ± 6.34 ^b^	21.50 ± 0.95 ^a^	6.24 ± 0.49 ^c^	3.11 ± 0.21 ^b^
AD_60_-VI/OH	95.03 ± 2.75 ^ef^	15.64 ± 0.93 ^cd^	1900.62 ± 100.27 ^c^	218.12 ± 5.47 ^c^	254.78 ± 5.35 ^c^	19.26 ± 0.73 ^a^	1.73 ± 0.15 ^b^	1.29 ± 0.07 ^a^
FD-VI/OH	47.64 ± 2.54 ^bcd^	18.25 ± 1.01 ^d^	1490.31 ± 110.71 ^b^	136.03 ± 4.24 ^b^	155.84 ± 4.24 ^b^	36.08 ± 2.07 ^b^	13.44 ± 0.61 ^e^	12.58 ± 0.83 ^c^

Results are presented as mean ± standard deviation. TPC—total phenolic content. TFC—total flavonoid content. TMA—total monomeric anthocyanin content. DPPH—2,2-diphenyl-1-picrylhydrazyl. FRAP—ferric reducing antioxidant power. GAE—gallic acid equivalent. CE—catechin equivalent. C3G—cyanidin 3-glucoside equivalent. TE—Trolox equivalent. L*—darkness–lightness. a*—green–red axis. b*—blue–yellow axis. (-)—not applicable. DM—dry matter. ^a–h^ Different lowercase letters in the same column indicate statistical difference (*p* ≤ 0.05), according to the LSD test. PS treated by vacuum impregnation + ohmic heating (VI/OH) and non-dried (PS-VI/OH) and dried by freeze-drying (FD-VI/OH) and air-drying at 40, 50, and 60 °C (AD_40_-VI/OH, AD_50_-VI/OH, and AD_60_-VI/OH). Control samples: PS non-treated by VI/OH and non-dried (PS-non-VI/OH) and dried by freeze-drying (FD-non-VI/OH) and air-drying at 40, 50, and 60 °C (AD_40_-non-VI/OH, AD_50_-non-VI/OH, and AD_60_-non-VI/OH).

**Table 2:** Individual phenolic compounds of blueberry juice and pear slices (PS) impregnated and non-impregnated with blueberry juice before and after the drying processes.

Phenolic Compound	Blueberry Juice (mg.100 mL^−1^)	PS (mg.100 g^−1^ Dry Matter)									
		PS-Non-VI/OH	AD_40_-Non-VI/OH	AD_50_-Non-VI/OH	AD_60_-Non-VI/OH	FD-Non-VI/OH	PS-VI/OH	AD_40_-VI/OH	AD_50_-VI/OH	AD_60_-VI/OH	FD-VI/OH
*Anthocyanin*											
Pelargonin	62.22 ± 2.62	nd	nd	nd	nd	nd	nd	nd	nd	nd	nd
Cyanidin 3- glucosidase	3.24 ± 0.15	nd	nd	nd	nd	nd	nd	nd	nd	nd	nd
Petunidin 3- glucosidase	6.83 ± 0.31	nd	nd	nd	nd	nd	nd	nd	nd	nd	nd
Cyanidin	23.55 ± 1.09	nd	nd	nd	nd	nd	20.18 ± 0.90 ^b^	0.76 ± 0.05 ^a^	0.69 ± 0.03 ^a^	0.56 ± 0.03 ^a^	0.87 ± 0.04 ^a^
Malvidin	nd	nd	nd	nd	nd	nd	nd	nd	nd	nd	nd
*Non-anthocyanin flavonoid*											
Epigallocatechin	125.47 ± 5.65	169.98 ± 5.90 ^f^	34.10 ± 1.51 ^bc^	35.86 ± 1.46 ^bc^	40.91 ± 1.75 ^c^	11.33 ± 0.58 ^a^	347.92 ± 15.95 ^g^	62.39 ± 2.97 ^d^	58.32 ± 2.45 ^d^	80.48 ± 3.69 ^e^	27.36 ± 1.27 ^b^
Catechin	18.49 ± 1.02	35.69 ± 1.60 ^d^	24.13 ± 1.09 ^ab^	25.44 ± 0.91 ^b^	31.01 ± 1.43 ^c^	23.49 ± 0.89 ^ab^	68.43 ± 2.82 ^e^	22.27 ± 0.95 ^a^	25.38 ± 0.90 ^b^	31.89 ± 1.60 ^c^	25.12 ± 1.08 ^b^
Epigallocatechin gallate	nd	nd	nd	nd	nd	nd	nd	nd	nd	nd	nd
Epicatechin	30.81 ± 1.50	27.48 ± 1.41 ^g^	5.53 ± 0.28 ^c^	5.63 ± 0.27 ^c^	6.61 ± 0.30 ^d^	7.75 ± 0.29 ^e^	9.51 ± 0.47 ^f^	2.58 ± 0.12 ^a^	2.64 ± 0.11 ^ab^	3.05 ± 0.14 ^ab^	4.51 ± 0.17 ^c^
Myricetin	8.84 ± 0.45	nd	nd	nd	nd	nd	9.98 ± 0.39 ^c^	2.14 ± 0.09 ^a^	2.00 ± 0.10 ^a^	1.91 ± 0.08 ^a^	2.62 ± 0.10 ^b^
Quercetin	2.28 ± 0.10	nd	0.36 ± 0.02 ^a^	0.48 ± 0.02 ^b^	0.55 ± 0.03 ^c^	0.35 ± 0.02 ^a^	nd	0.66 ± 0.03 ^d^	0.75 ± 0.03 ^e^	0.96 ± 0.05 ^f^	0.67 ± 0.03 ^d^
*Phenolic acid*											
Gallic acid	3.31 ± 0.14	8.08 ± 0.30 ^g^	7.70 ± 0.25 ^f^	7.24 ± 0.22 ^e^	6.69 ± 0.27 ^d^	7.01 ± 0.22 ^de^	4.73 ± 0.16 ^c^	4.36 ± 0.15 ^b^	4.18 ± 0.16 ^b^	3.80 ± 0.14 ^a^	4.01 ± 0.15 ^ab^
Caffeic acid	5.17 ± 0.24	2.44 ± 0.11 ^e^	0.85 ± 0.03 ^c^	0.87 ± 0.04 ^c^	0.93 ± 0.04 ^c^	0.90 ± 0.04 ^c^	1.75 ± 0.07 ^d^	0.53 ± 0.02 ^a^	0.59 ± 0.03 ^ab^	0.62 ± 0.03 ^b^	0.59 ± 0.03 ^ab^
*p*-Coumaric acid	3.04 ± 0.14	nd	nd	nd	nd	nd	nd	nd	nd	nd	nd
*Sum*	293.25 ± 7.45	243.68 ± 4.88	72.68 ± 2.50	75.52 ± 2.73	86.69 ± 2.97	50.83 ± 1.88	462.49 ± 18.53	95.70 ± 3.97	94.56 ± 3.37	123.27 ± 4.69	65.74 ± 2.56

Results are presented as mean ± standard deviation. nd—not detected. ^a–g^ Different lowercase letters in the same row, for PS, indicate statistical difference (*p* ≤ 0.05) according to the LSD test. PS treated by vacuum impregnation + ohmic heating (VI/OH) and non-dried (PS-VI/OH) and dried by freeze-drying (FD-VI/OH) and air-drying at 40, 50, and 60 °C (AD_40_-VI/OH, AD_50_-VI/OH, and AD_60_-VI/OH). Control samples: PS non-treated by VI/OH and non-dried (PS-non-VI/OH) and dried by freeze-drying (FD-non-VI/OH) and air-drying at 40, 50, and 60 °C (AD_40_-non-VI/OH, AD_50_-non-VI/OH, and AD_60_-non-VI/OH).

## Data Availability

Not applicable.

## Supplementary Materials

The following supporting information can be downloaded at: https://www.mdpi.com/article/10.3390/antiox12071408/s1, Table S1: Methodologies of total bioactive compounds, antioxidant capacity, and individual phenolic compounds; Table S2: Total bioactive compounds and antioxidant capacity of blueberry juice treated by ohmic heating throughout 20 min at 30, 40, and 50 °C; Table S3: Vacuum impregnation assisted by ohmic heating kinetics of pear slices enriched with blueberry juice throughout 180 min at 30, 40, and 50 °C on total phenolic content (TPC), total flavonoid content (TFC), and total monomeric anthocyanin content (TMA); Table S4: Vacuum impregnation assisted by ohmic heating kinetics of pear slices enriched with blueberry juice throughout 180 min at 30, 40, and 50 °C on antioxidant capacity (µmol TE.100 g^−1^ dry matter).
