# Supplementary material for: Processing of Enriched Pear Slices with Blueberry Juice: Phenolics, Antioxidant, and Color Characteristics
Source: Antioxidants (Basel). 2023 Jul 11;12(7):1408. doi: 10.3390/antiox12071408 (PMC10376512; doi:10.3390/antiox12071408)
Supplement: Supplementary file 1 [file antioxidants-12-01408-s001.zip › Table S1.pdf]

Table S1: Methodologies of total bioactive compounds, antioxidant capacity, and individual phenolic compounds.

Methodologies	Description
Total phenolic content	Sample (100 µL) was mixed with distilled water (7.9 mL) and Folin-Ciocalteu reagent (500 µL). After 1 to 8 min, 1.5 mL of 20% (w/v) $NaHCO_3$ was added to the mixture and incubated for 2 h in the dark. Absorbance was measured at 760 nm in a spectrophotometer (Genesys 10 S UV-Vis, Thermo Scientific, Germany). Result was deducted from the blank and expressed as mg of gallic acid equivalent (GAE) per 100 mL or per 100 g of dry matter. Further dilutions were performed when necessary.
Total flavonoid content	Sample (250 µL) was mixed with distilled water (1250 µL) and 75 µL of 5% (w/v) $NaNO_2$. After 6 min, 150 µL of 10% (w/v) $AlCl_3.6H_2O$ was added to the mixture. After 5 min, 500 µL of 1 M NaOH and 250 µL of distilled water were added to the mixture. Absorbance was immediately measured at 510 nm. Result was deducted from the blank and expressed as mg of catechin equivalent (CE) per 100 mL or per 100 g of dry matter. Further dilutions were performed when necessary.
Total monomeric anthocyanin content	An aliquot of 0.5 mL of sample was mixed with 2 mL of 0.025 M KCl (pH 1.0). This same procedure was performed with 2 mL of 0.4 M $CH_3COONa.3H_2O$ (pH 4.5). After 25 min in the dark, the absorbance of the mixtures was measured at 510 nm and 700 nm. Results were deducted from the blank and expressed as µg cyanidin 3-glucoside equivalent (C3G) per 100 mL or per 100 g of dry matter. Further dilutions were performed when necessary.
DPPH (2,2-diphenyl-1-picrylhydrazyl) free radical scavenging	An aliquot of 150 µL of the sample was mixed with 2850 µL of DPPH methanolic solution adjusted to 1.1 absorbance units. After 20 min of incubation in the dark, the absorbance of the solution was measured at 515 nm. Result as deducted from the blank and expressed as µmol Trolox equivalents (TE) per 100 mL or per 100 g of dry matter. Further dilutions were performed when necessary.
Ferric reducing antioxidant power (FRAP)	FRAP reagent was prepared freshly by mixing 25 mL of 300 mM $CH_3COONa.3H_2O$ (pH 3.6), 2.5 mL of 10 mM 2,4,6-Tris(2-pyridyl)-s-triazine (in 40 mM HCl), and 2.5 mL of 20 mM $FeCl_3.6H_2O$. This solution was warmed at 37 °C before use. Briefly, 150 µL of the sample was mixed with 2850 µL of FRAP reagent. After 30 min of incubation in the dark and at 37 °C, the absorbance was measured at 593 nm. Result was deducted from the blank and expressed as µmol TE per 100 mL or per 100 g of dry matter. Further dilutions were performed when necessary.
Phenolic compounds by high-performance liquid chromatography	Chromatography separation was carried out in a Purospher STAR RP-18 endcapped column (5 µm, 125 x 4 mm; Merck, Germany) at 40 °C, with 0.8 $mL.min^{-1}$ flow rate, and a gradient of mobile phase (solvent A - water/acetonitrile/formic acid at the proportion of 8.7:0.3:1, v/v/v); solvent B - water/acetonitrile/formic acid at the proportion of 4:5:1, v/v/v) of 94–70% of solvent A (0–15 min), 70–50% of solvent A (16–30 min), 50–40% of solvent A (31–35 min), 40–94% of solvent A (36–41 min), and 94% of solvent A for stabilization (41–45 min). Phenolic compounds were monitored at 280 nm for phenolic acids and non-anthocyanin flavonoids, and at 520 nm for anthocyanins. Results were expressed as mg per 100 mL or per 100 g of dry matter. Further dilutions were performed when necessary.
