# Supplementary material for: Processing of Enriched Pear Slices with Blueberry Juice: Phenolics, Antioxidant, and Color Characteristics
Source: Antioxidants (Basel). 2023 Jul 11;12(7):1408. doi: 10.3390/antiox12071408 (PMC10376512; doi:10.3390/antiox12071408)
Supplement: Supplementary file 1 [file antioxidants-12-01408-s001.zip › Table S2.pdf]

Table S2: Total bioactive compounds and antioxidant capacity of blueberry juice treated by ohmic heating throughout 20 min at 30, 40, and 50 °C.

Temperature (°C)	Time (min)	TPC (mg GAE.100 mL^{-1})	TFC (mg CE.100 mL^{-1})	TMA (µg C3G.100 mL^{-1})	Antioxidant capacity	
					DPPH (µmol TE.100 mL^{-1})	FRAP (µmol TE.100 mL^{-1})
Non-treated juice		152.00 ± 3.14 ^{a}	90.93 ± 0.50 ^{a}	354.03 ± 9.23 ^{a}	327.94 ± 5.54 ^{a}	403.84 ± 0.84 ^{a}
30	5	156.54 ± 3.27 ^{ab}	97.45 ± 1.09 ^{b}	473.15 ± 9.64 ^{b}	343.77 ± 5.02 ^{b}	403.24 ± 2.80 ^{a}
	10	161.69 ± 4.48 ^{b}	96.98 ± 3.76 ^{b}	642.35 ± 11.23 ^{d}	371.00 ± 7.94 ^{c}	409.77 ± 1.66 ^{ab}
	15	174.12 ± 2.28 ^{cd}	124.49 ± 0.41 ^{e}	1074.72 ± 19.42 ^{hi}	419.88 ± 3.15 ^{e}	428.97 ± 7.40 ^{c}
	20	188.97 ± 6.44 ^{f}	134.46 ± 1.64 ^{g}	1219.04 ± 23.26 ^{j}	430.16 ± 2.35 ^{e}	464.97 ± 7.52 ^{e}
40	5	175.93 ± 2.28 ^{cd}	110.02 ± 1.64 ^{c}	512.53 ± 19.26 ^{c}	335.16 ± 9.46 ^{ab}	425.04 ± 7.07 ^{bc}
	10	178.36 ± 1.28 ^{de}	118.09 ± 2.49 ^{d}	826.34 ± 11.53 ^{e}	365.44 ± 7.51 ^{c}	430.84 ± 8.00 ^{c}
	15	179.27 ± 2.57 ^{de}	122.83 ± 2.05 ^{e}	1024.12 ± 34.24 ^{g}	366.27 ± 5.91 ^{c}	438.17 ± 9.38 ^{cd}
	20	182.30 ± 2.28 ^{e}	136.59 ± 1.48 ^{gh}	1085.53 ± 16.13 ^{i}	382.11 ± 9.14 ^{d}	449.10 ± 9.20 ^{de}
50	5	170.78 ± 2.09 ^{c}	118.32 ± 3.96 ^{d}	442.53 ± 11.53 ^{b}	330.72 ± 4.73 ^{a}	428.04 ± 15.95 ^{c}
	10	172.90 ± 0.90 ^{cd}	129.71 ± 1.06 ^{f}	969.23 ± 28.24 ^{f}	361.83 ± 9.27 ^{c}	448.44 ± 19.06 ^{de}
	15	195.03 ± 6.05 ^{g}	148.57 ± 0.50 ^{i}	1040.42 ± 19.53 ^{gh}	392.38 ± 4.88 ^{d}	519.10 ± 12.23 ^{f}
	20	201.09 ± 1.57 ^{h}	139.43 ± 1.48 ^{h}	1035.14 ± 16.35 ^{g}	427.11 ± 2.09 ^{e}	562.24 ± 3.67 ^{g}

Results are presented as mean ± standard deviation. TPC – total phenolic content. TFC – total flavonoid content. TMA – total monomeric anthocyanins. DPPH – 2,2-diphenyl-1-picrylhydrazyl. FRAP – ferric reducing antioxidant power. GAE – gallic acid equivalent. CE – catechin equivalent. C3G – cyanidin 3-glucoside equivalent. TE – Trolox equivalent. $^{a–j}$ Different lowercase letters in the same column indicate statistical difference ($p \leq 0.05$) according to the LSD test.
