# Supplementary material for: Processing of Enriched Pear Slices with Blueberry Juice: Phenolics, Antioxidant, and Color Characteristics
Source: Antioxidants (Basel). 2023 Jul 11;12(7):1408. doi: 10.3390/antiox12071408 (PMC10376512; doi:10.3390/antiox12071408)
Supplement: Supplementary file 1 [file antioxidants-12-01408-s001.zip › Table S3.pdf]

Table S3: Vacuum impregnation assisted by ohmic heating kinetics of pear slices enriched with blueberry juice throughout 180 min at 30, 40, and 50 °C on total phenolic content (TPC), total flavonoid content (TFC), and total monomeric anthocyanins (TMA).

**Time (min)**	**TPC (mg GAE.100 g^{-1} dry matter)**			**TFC (mg CE.100 g^{-1} dry matter)**			**TMA (µg C3G.100 g^{-1} dry matter)**		
	**30 °C**	**40 °C**	**50 °C**	**30 °C**	**40 °C**	**50 °C**	**30 °C**	**40 °C**	**50 °C**
Non-treated pear	196.33 ± 4.43 ^{e,A}	196.33 ± 4.43 ^{d,A}	196.33 ± 4.43 ^{d,A}	47.71 ± 0.49 ^{e,A}	47.71 ± 0.49 ^{de,A}	47.71 ± 0.49 ^{c,A}	-	-	-
5	189.75 ± 5.78 ^{de,B}	162.90 ± 0.17 ^{bc,A}	152.07 ± 6.07 ^{a,A}	17.07 ± 0.31 ^{a,A}	29.16 ± 0.46 ^{a,B}	29.75 ± 0.80 ^{a,B}	129.13 ± 1.89 ^{a,A}	261.40 ± 3.01 ^{a,B}	125.25 ± 1.17 ^{a,A}
10	141.78 ± 3.57 ^{a,A}	151.32 ± 2.97 ^{a,B}	160.44 ± 0.54 ^{ab,C}	19.46 ± 0.14 ^{a,A}	33.50 ± 1.57 ^{b,B}	36.02 ± 0.43 ^{ab,B}	197.08 ± 2.10 ^{b,A}	309.66 ± 4.71 ^{a,C}	246.16 ± 8.46 ^{b,B}
15	150.37 ± 5.17 ^{a,A}	147.02 ± 3.71 ^{a,A}	172.04 ± 4.88 ^{bc,B}	23.50 ± 0.92 ^{b,A}	32.48 ± 1.40 ^{b,B}	39.05 ± 1.35 ^{b,C}	204.19 ± 2.35 ^{b,A}	517.93 ± 9.09 ^{b,C}	389.26 ± 5.10 ^{c,B}
20	177.22 ± 7.54 ^{bc,B}	155.72 ± 7.41 ^{ab,A}	175.90 ± 5.66 ^{c,B}	36.50 ± 0.99 ^{c,A}	38.59 ± 0.43 ^{c,A}	55.81 ± 1.11 ^{d,B}	263.42 ± 4.42 ^{c,A}	637.35 ± 24.62 ^{c,C}	431.28 ± 3.87 ^{c,B}
25	182.81 ± 7.20 ^{cd,B}	169.27 ± 1.17 ^{c,A}	181.43 ± 7.22 ^{c,AB}	42.80 ± 0.86 ^{d,A}	43.96 ± 1.68 ^{d,A}	73.19 ± 0.30 ^{e,B}	331.92 ± 13.10 ^{d,A}	900.09 ± 21.87 ^{d,C}	670.49 ± 14.09 ^{d,B}
30	187.72 ± 6.55 ^{de,A}	195.83 ± 3.09 ^{d,A}	184.15 ± 2.72 ^{c,A}	43.12 ± 1.57 ^{d,A}	50.06 ± 1.62 ^{ef,B}	90.78 ± 3.39 ^{f,C}	395.71 ± 18.49 ^{e,A}	1200.95 ± 13.30 ^{e,B}	1186.81 ± 38.64 ^{e,B}
45	171.00 ± 2.27 ^{b,A}	208.76 ± 2.37 ^{e,B}	218.34 ± 7.75 ^{e,B}	47.99 ± 1.86 ^{e,A}	53.06 ± 1.77 ^{f,A}	136.99 ± 3.07 ^{g,B}	439.69 ± 7.42 ^{f,A}	1282.06 ± 16.85 ^{f,B}	2264.71 ± 64.01 ^{f,C}
60	176.15 ± 0.98 ^{bc,A}	192.95 ± 2.46 ^{d,B}	351.81 ± 0.90 ^{f,C}	59.90 ± 1.52 ^{f,A}	90.48 ± 4.28 ^{g,B}	167.48 ± 4.42 ^{h,C}	735.81 ± 22.86 ^{g,A}	1209.87 ± 42.12 ^{e,B}	2665.47 ± 10.18 ^{i,C}
75	257.96 ± 5.45 ^{g,B}	231.35 ± 5.85 ^{f,A}	349.79 ± 6.34 ^{f,C}	71.24 ± 2.16 ^{g,A}	113.43 ± 1.03 ^{h,B}	164.64 ± 5.86 ^{h,C}	924.64 ± 32.67 ^{h,A}	1576.31 ± 36.70 ^{g,B}	2401.34 ± 63.14 ^{g,C}
90	253.47 ± 1.67 ^{g,A}	239.07 ± 10.91^{fg,A}	398.03 ± 8.97 ^{i,B}	101.38 ± 3.33 ^{h,A}	134.79 ± 2.56 ^{j,B}	182.62 ± 4.81 ^{j,C}	1194.36 ± 31.66 ^{k,A}	2282.22 ± 84.48 ^{i,B}	2489.15 ± 58.84 ^{h,C}
120	193.14 ± 3.93 ^{e,A}	248.24 ± 6.26 ^{g,B}	365.48 ± 7.35 ^{g,C}	98.25 ± 1.67 ^{h,A}	129.80 ± 3.01 ^{i,B}	175.20 ± 6.29 ^{i,C}	1116.66 ± 42.45 ^{j,A}	2035.41 ± 35.59 ^{h,B}	2605.87 ± 84.35 ^{i,C}
180	205.97 ± 2.91 ^{f,A}	240.66 ± 8.92 ^{fg,B}	378.62 ± 9.65 ^{h,C}	106.66 ± 2.83 ^{i,A}	154.56 ± 1.57 ^{k,B}	177.52 ± 5.10 ^{ij,C}	1004.72 ± 38.62 ^{i,A}	2587.58 ± 29.52 ^{j,B}	3258.69 ± 58.02 ^{j,C}

Results are presented as mean ± standard deviation. GAE – gallic acid equivalent. CE – catechin equivalent. C3G – cyanidin 3-glucoside equivalent. (-) not applicable. $^{a–k}$ Different lowercase letters in the same column indicate statistical difference ($p \leq 0.05$) according to the LSD test. $^{A–C}$ Different capital letters in the same row for each assay, indicate statistical difference ($p \leq 0.05$) according to the LSD test.
