# Supplementary material for: Processing of Enriched Pear Slices with Blueberry Juice: Phenolics, Antioxidant, and Color Characteristics
Source: Antioxidants (Basel). 2023 Jul 11;12(7):1408. doi: 10.3390/antiox12071408 (PMC10376512; doi:10.3390/antiox12071408)
Supplement: Supplementary file 1 [file antioxidants-12-01408-s001.zip › Table S4.pdf]

Table S4: Vacuum impregnation assisted by ohmic heating kinetics of pear slices enriched with blueberry juice throughout 180 min at 30, 40, and 50 °C on antioxidant capacity (µmol TE.100 g^{-1} dry matter).

Time (min)	DPPH			FRAP		
	**30 °C**	**40 °C**	**50 °C**	**30 °C**	**40 °C**	**50 °C**
Non-treated pear	630.53 ± 3.62 ^{c,A}	630.53 ± 3.62 ^{d,A}	630.53 ± 3.62 ^{c,A}	619.54 ± 11.53 ^{e,A}	619.54 ± 11.53 ^{f,A}	619.54 ± 11.53 ^{d,A}
5	534.67 ± 6.54 ^{a,C}	471.35 ± 3.56 ^{a,B}	454.74 ± 9.53 ^{a,A}	402.42 ± 5.35 ^{a,B}	371.43 ± 7.45 ^{a,A}	401.76 ± 5.23 ^{a,B}
10	521.24 ± 11.25 ^{a,B}	478.74 ± 18.54 ^{ab,A}	476.35 ± 20.64 ^{a,A}	402.73 ± 3.73 ^{a,A}	407.53 ± 3.84 ^{b,A}	443.24 ± 11.23 ^{b,B}
15	549.42 ± 7.63 ^{a,B}	499.35 ± 10.45 ^{ab,A}	576.19 ± 18.45 ^{b,C}	416.35 ± 16.53 ^{ab,A}	427.54 ± 3.35 ^{bc,A}	535.74 ± 2.34 ^{c,B}
20	545.56 ± 19.54 ^{a,AB}	515.74 ± 13.45 ^{bc,A}	553.24 ± 15.74 ^{b,B}	432.74 ± 5.63 ^{b,A}	447.45 ± 12.64 ^{c,A}	794.05 ± 11.35 ^{e,B}
25	584.86 ± 22.74 ^{b,B}	548.73 ± 6.63 ^{c,A}	624.74 ± 20.02 ^{c,C}	435.63 ± 10.63 ^{bc,A}	488.24 ± 11.35 ^{d,B}	833.34 ± 5.36 ^{f,C}
30	617.92 ± 21.34 ^{bc,A}	656.43 ± 19.45 ^{d,A}	730.93 ± 21.03 ^{d,B}	456.23 ± 11.73 ^{c,A}	561.63 ± 17.36 ^{e,B}	994.56 ± 10.64 ^{g,C}
45	644.23 ± 26.75 ^{c,A}	718.03 ± 4.73 ^{e,B}	987.93 ± 12.64 ^{e,C}	499.92 ± 7.34 ^{d,A}	702.02 ± 27.74 ^{h,B}	1111.82 ± 35.54 ^{h,C}
60	638.73 ± 29.42 ^{c,A}	750.82 ± 25.34 ^{e,B}	974.55 ± 7.95 ^{e,C}	615.03 ± 11.03 ^{e,A}	663.06 ± 9.34 ^{g,A}	1377.45 ± 30.63 ^{k,B}
75	746.35 ± 11.02 ^{d,A}	903.35 ± 18.12 ^{f,B}	998.63 ± 12.64 ^{e,C}	781.53 ± 19.85 ^{h,B}	702.34 ± 24.53 ^{h,A}	1222.34 ± 16.35 ^{i,C}
90	747.93 ± 3.24 ^{d,A}	952.30 ± 3.63 ^{g,B}	1061.83 ± 6.63 ^{f,C}	741.46 ± 2.39 ^{g,A}	886.66 ± 28.13 ^{j,B}	1278.64 ± 24.35 ^{j,C}
120	756.82 ± 10.86 ^{d,A}	1046.26 ± 30.20 ^{h,B}	1044.92 ± 22.73 ^{f,B}	672.14 ± 5.98 ^{f,A}	743.25 ± 6.84 ^{i,B}	1204.35 ± 5.63 ^{i,C}
180	755.64 ± 36.92 ^{d,A}	1197.11 ± 58.64 ^{i,B}	1109.44 ± 19.46 ^{g,B}	813.63 ± 26.34 ^{i,A}	1124.45 ± 32.35 ^{k,B}	1278.34 ± 13.36 ^{j,C}

Results are presented as mean ± standard deviation. DPPH – 2,2-diphenyl-1-picrylhydrazyl. FRAP – ferric reducing antioxidant power. TE – Trolox equivalent. $^{a–k}$ Different lowercase letters in the same column indicate statistical difference ($p \leq 0.05$) according to the LSD test. $^{A–C}$ Different capital letters in the same row for each assay, indicate statistical difference ($p \leq 0.05$) according to the LSD test.
